# Chlorido[*N*,*N*′-dibenzyl-*N*,*N*′-bis­(pyridin-2-ylmeth­yl)ethane-1,2-diamine]­copper(II) perchlorate methanol monosolvate

**DOI:** 10.1107/S1600536812017941

**Published:** 2012-04-28

**Authors:** Hui-Ting Song, Jia-Wei Mao, Ming Liu, Zhi-Quan Pan

**Affiliations:** a Key Laboratory for Green Chemical Process of the Ministry of Education, Wuhan Institute of Technology, Wuhan 430073, People’s Republic of China; b College of Chemistry and Molecular Sciences, Wuhan University, Wuhan 430072, People’s Republic of China

## Abstract

In the title solvated mol­ecular salt, [CuCl(C_28_H_30_N_4_)]ClO_4_·CH_3_OH, the Cu^2+^ ion is coordinated by the *N*,*N*′,*N*′′,*N*′′′-tetra­dentate ligand and a chloride ion, generating a very distorted square-based pyramidal CuN_4_Cl coordination geometry with the Cl^−^ ion in the basal position. In the crystal, the solvent mol­ecules and anions are linked by weak O—H⋯O hydrogen bonding.

## Related literature

For related copper complexes, see: Cejudo *et al.* (2006 ▶); Vaidyanathan & Nair (2003 ▶); Wang *et al.* (2007 ▶); Xiao *et al.* (2011 ▶). For further synthetic details, see: Hamid & Hamid (2010 ▶); Fenton *et al.* (1995 ▶); Sun *et al.* (2002 ▶). For geometric descriptors of five-coordinate metal ions, see: Addison *et al.* (1984 ▶).
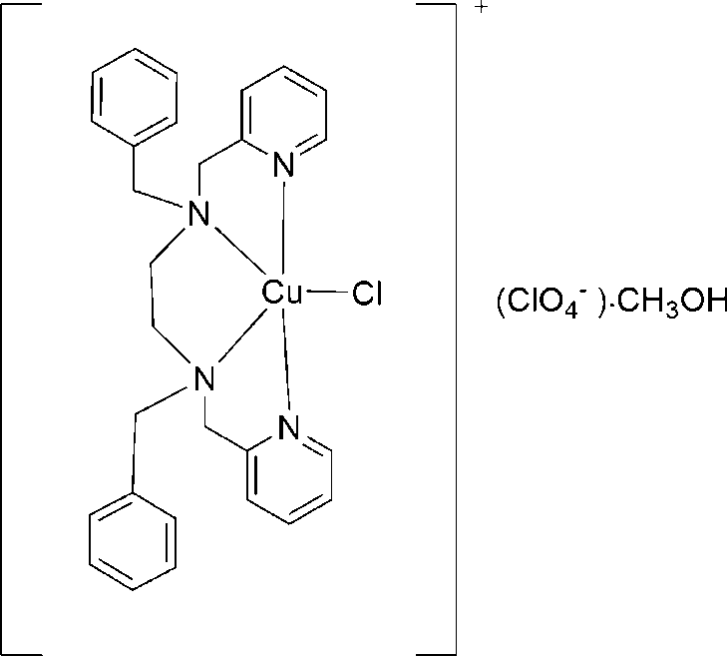


## Experimental

### 

#### Crystal data

[CuCl(C_28_H_30_N_4_)]ClO_4_·CH_4_O
*M* *_r_* = 653.04
Monoclinic, 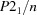

*a* = 17.800 (2) Å
*b* = 10.5804 (13) Å
*c* = 18.107 (2) Å
β = 118.374 (1)°
*V* = 3000.3 (6) Å^3^
*Z* = 4
Mo *K*α radiation
μ = 0.95 mm^−1^
*T* = 291 K
0.28 × 0.24 × 0.22 mm

#### Data collection

Bruker SMART APEX CCD diffractometer
Absorption correction: multi-scan (*SADABS*; Bruker, 2000 ▶) *T*_min_ = 0.777, *T*_max_ = 0.818
16310 measured reflections
5885 independent reflections
4329 reflections with *I* > 2σ(*I*)
*R*_int_ = 0.042

#### Refinement

*R*[*F* ^2^ > 2σ(*F* ^2^)] = 0.047
*wR*(*F* ^2^) = 0.110
*S* = 1.04
5885 reflections
372 parameters
H-atom parameters constrained
Δρ_max_ = 0.24 e Å^−3^
Δρ_min_ = −0.36 e Å^−3^

### 

Data collection: *SMART* (Bruker, 2000 ▶); cell refinement: *SAINT* (Bruker, 2000 ▶); data reduction: *SAINT*; program(s) used to solve structure: *SHELXTL* (Sheldrick, 2008 ▶); program(s) used to refine structure: *SHELXTL*; molecular graphics: *SHELXTL*; software used to prepare material for publication: *SHELXTL*.

## Supplementary Material

Crystal structure: contains datablock(s) global, I. DOI: 10.1107/S1600536812017941/hb6747sup1.cif

Structure factors: contains datablock(s) I. DOI: 10.1107/S1600536812017941/hb6747Isup2.hkl

Additional supplementary materials:  crystallographic information; 3D view; checkCIF report

## Figures and Tables

**Table 1:** Selected bond lengths (Å)

Cu1—N3	1.983 (3)
Cu1—N2	2.022 (2)
Cu1—N4	2.087 (2)
Cu1—N1	2.152 (3)
Cu1—Cl2	2.2830 (9)

**Table 2:** Hydrogen-bond geometry (Å, °)

*D*—H⋯*A*	*D*—H	H⋯*A*	*D*⋯*A*	*D*—H⋯*A*
O1—H1*B*⋯O12	0.96	2.48	3.140 (4)	126

**Fig. 1.**
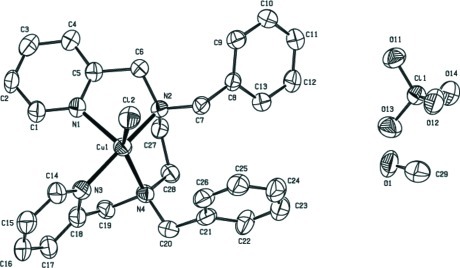
Molecular structure of the title compound. Displacement ellipsoids are drawn at the 30% probability level. H atoms have been omitted for clarity.

## supplementary crystallographic information

### Comment

Recently, study of copper complex with polynitrogen ligands has been given considerable attention because of their interesting biochemical properties (Cejudo *et al.*, 2006; Vaidyanathan *et al.*, 2003; Wang *et al.*, 2007; Xiao *et al.*, 2011). In this paper, we report on the crystal structure of a new copper(II) complex with the polynitrogen ligand N^1^,N^2^-dibenzyl-N^1^,N^2^-bis(pyridin-2-ylmethyl)ethane-1,2-diamine (*L*).

The structure of the title compound is shown in Fig.1. The Cu(II) atom is five-coordinated by two amino N atoms and two pyridine N atoms from the ligand *L* and one Cl atom. The coordination geometry for central Cu(II) atom can be described as distorted square based pyramidal, with τ = 0.30 (Addison *et al.*, 1984).

### Experimental

*N*,*N*'-bis(2-benzylmethyl)-1,2-diaminoethane was prepared using a variant of the method suggested by Hamid & Hamid (2010). The ligand *L* was synthesized according to a procedure reported previously (Fenton *et al.* 1995; Sun *et al.*, 2002). To a refluxing solution of *L* (0.149 g, 0.3 mmol) in absolute methanol (15 ml) was added dropwise a solution of CuCl_2_ (0.0404 g 0.3 mmol)and Cu(ClO_4_)_2_.6H_2_O (0.112 g, 0.3 mmol). After the addition was completed, the resulting solution became green, and the mixture was stirred for about 6 h at room temperature. After filtration, blue blocks were obtained by slow evaporation of the absolute methanol solution at room temperature for two weeks.

### Refinement

All C-bound H atoms were placed in calculated positions with 0.93–0.97 Å, and included in the refinement in the riding-model approximation, with *U*(H) set to 1.2–1.5*U*_eq_(C).

### Crystal data

**Table:**

[CuCl(C_28_H_30_N_4_)]ClO_4_·CH_4_O	*F*(000) = 1356
*M**_r_* = 653.04	*D*_x_ = 1.446 Mg m^−^^3^
Monoclinic, *P*2_1_/*n*	Mo *K*α radiation, λ = 0.71073 Å
Hall symbol: -P 2yn	Cell parameters from 4897 reflections
*a* = 17.800 (2) Å	θ = 2.2–24.4°
*b* = 10.5804 (13) Å	µ = 0.95 mm^−^^1^
*c* = 18.107 (2) Å	*T* = 291 K
β = 118.374 (1)°	Block, blue
*V* = 3000.3 (6) Å^3^	0.28 × 0.24 × 0.22 mm
*Z* = 4	

### Data collection

**Table:**

Bruker SMART APEX CCD diffractometer	5885 independent reflections
Radiation source: sealed tube	4329 reflections with *I* > 2σ(*I*)
Graphite monochromator	*R*_int_ = 0.042
phi and ω scans	θ_max_ = 26.0°, θ_min_ = 2.2°
Absorption correction: multi-scan (*SADABS*; Bruker, 2000)	*h* = −21→21
*T*_min_ = 0.777, *T*_max_ = 0.818	*k* = −13→12
16310 measured reflections	*l* = −22→15

### Refinement

**Table:**

Refinement on *F*^2^	Primary atom site location: structure-invariant direct methods
Least-squares matrix: full	Secondary atom site location: difference Fourier map
*R*[*F*^2^ > 2σ(*F*^2^)] = 0.047	Hydrogen site location: inferred from neighbouring sites
*wR*(*F*^2^) = 0.110	H-atom parameters constrained
*S* = 1.04	*w* = 1/[σ^2^(*F*_o_^2^) + (0.05*P*)^2^ + 1.22*P*] where *P* = (*F*_o_^2^ + 2*F*_c_^2^)/3
5885 reflections	(Δ/σ)_max_ = 0.001
372 parameters	Δρ_max_ = 0.24 e Å^−^^3^
0 restraints	Δρ_min_ = −0.36 e Å^−^^3^

### Special details

**Table:**

Geometry. All esds (except the esd in the dihedral angle between two l.s. planes) are estimated using the full covariance matrix. The cell esds are taken into account individually in the estimation of esds in distances, angles and torsion angles; correlations between esds in cell parameters are only used when they are defined by crystal symmetry. An approximate (isotropic) treatment of cell esds is used for estimating esds involving l.s. planes.
Refinement. Refinement of F^2^ against ALL reflections. The weighted *R*-factor *wR* and goodness of fit S are based on F^2^, conventional *R*-factors *R* are based on F, with F set to zero for negative F^2^. The threshold expression of F^2^ > 2sigma(F^2^) is used only for calculating *R*-factors(gt) *etc*. and is not relevant to the choice of reflections for refinement. *R*-factors based on F^2^ are statistically about twice as large as those based on F, and R– factors based on ALL data will be even larger.

### Fractional atomic coordinates and isotropic or equivalent isotropic displacement parameters (Å^2^)

**Table:**

	*x*	*y*	*z*	*U*_iso_*/*U*_eq_	
C1	0.8517 (2)	−0.0164 (3)	0.6532 (2)	0.0510 (8)	
H1	0.8516	−0.0832	0.6199	0.061*	
C2	0.8816 (2)	−0.0377 (3)	0.7372 (2)	0.0506 (8)	
H2	0.9009	−0.1173	0.7601	0.061*	
C3	0.8824 (2)	0.0609 (4)	0.7866 (2)	0.0529 (9)	
H3	0.9028	0.0492	0.8439	0.064*	
C4	0.8530 (2)	0.1772 (3)	0.7508 (2)	0.0476 (8)	
H4	0.8530	0.2451	0.7834	0.057*	
C5	0.82358 (18)	0.1917 (3)	0.66583 (18)	0.0356 (6)	
C6	0.7922 (2)	0.3167 (3)	0.62251 (18)	0.0400 (7)	
H6A	0.7611	0.3600	0.6469	0.048*	
H6B	0.8408	0.3688	0.6318	0.048*	
C7	0.72963 (18)	0.4225 (3)	0.48671 (18)	0.0364 (6)	
H7A	0.6993	0.4058	0.4269	0.044*	
H7B	0.7869	0.4491	0.5004	0.044*	
C8	0.68568 (18)	0.5318 (3)	0.50492 (18)	0.0360 (6)	
C9	0.7309 (2)	0.6120 (3)	0.5716 (2)	0.0436 (7)	
H9	0.7882	0.5960	0.6083	0.052*	
C10	0.6910 (2)	0.7169 (3)	0.5842 (2)	0.0499 (8)	
H10	0.7217	0.7699	0.6298	0.060*	
C11	0.6071 (2)	0.7426 (3)	0.5301 (2)	0.0515 (8)	
H11	0.5811	0.8134	0.5384	0.062*	
C12	0.5617 (2)	0.6636 (3)	0.4638 (2)	0.0542 (9)	
H12	0.5045	0.6803	0.4274	0.065*	
C13	0.6003 (2)	0.5599 (3)	0.4508 (2)	0.0441 (7)	
H13	0.5689	0.5074	0.4051	0.053*	
C14	0.8694 (2)	−0.0635 (3)	0.4603 (2)	0.0523 (8)	
H14	0.9185	−0.0147	0.4794	0.063*	
C15	0.8721 (2)	−0.1880 (4)	0.4398 (2)	0.0550 (9)	
H15	0.9223	−0.2235	0.4451	0.066*	
C16	0.7996 (2)	−0.2578 (4)	0.4116 (2)	0.0547 (9)	
H16	0.8000	−0.3424	0.3979	0.066*	
C17	0.7244 (2)	−0.2037 (3)	0.4031 (2)	0.0512 (8)	
H17	0.6743	−0.2506	0.3824	0.061*	
C18	0.7261 (2)	−0.0808 (3)	0.4257 (2)	0.0440 (7)	
C19	0.65156 (19)	−0.0122 (3)	0.4241 (2)	0.0420 (7)	
H19A	0.5985	−0.0494	0.3823	0.050*	
H19B	0.6534	−0.0185	0.4784	0.050*	
C20	0.6269 (2)	0.1318 (3)	0.31094 (19)	0.0419 (7)	
H20A	0.6577	0.0699	0.2961	0.050*	
H20B	0.5667	0.1106	0.2805	0.050*	
C21	0.63972 (19)	0.2601 (3)	0.28309 (18)	0.0420 (7)	
C22	0.5718 (2)	0.3417 (4)	0.2408 (2)	0.0567 (9)	
H22	0.5167	0.3152	0.2263	0.068*	
C23	0.5853 (2)	0.4638 (4)	0.2196 (2)	0.0579 (9)	
H23	0.5395	0.5188	0.1921	0.070*	
C24	0.6649 (3)	0.5012 (4)	0.2394 (2)	0.0601 (10)	
H24	0.6740	0.5822	0.2254	0.072*	
C25	0.7326 (2)	0.4209 (4)	0.2799 (2)	0.0561 (9)	
H25	0.7873	0.4473	0.2927	0.067*	
C26	0.7200 (2)	0.3020 (4)	0.3015 (2)	0.0498 (8)	
H26	0.7665	0.2484	0.3293	0.060*	
C27	0.65133 (19)	0.2506 (3)	0.51552 (19)	0.0402 (7)	
H27A	0.6191	0.3162	0.5254	0.048*	
H27B	0.6598	0.1815	0.5539	0.048*	
C28	0.60207 (18)	0.2044 (3)	0.4264 (2)	0.0414 (7)	
H28A	0.5526	0.1569	0.4199	0.050*	
H28B	0.5819	0.2763	0.3887	0.050*	
C29	0.5166 (3)	0.8830 (4)	0.1346 (3)	0.0703 (11)	
H29A	0.4710	0.9427	0.1070	0.105*	
H29B	0.5199	0.8293	0.0935	0.105*	
H29C	0.5695	0.9276	0.1652	0.105*	
Cl1	0.39157 (5)	0.94576 (7)	0.32224 (5)	0.04209 (18)	
Cl2	0.90470 (5)	0.25490 (9)	0.50649 (6)	0.0526 (2)	
Cu1	0.78296 (2)	0.15945 (3)	0.49085 (2)	0.03736 (12)	
N1	0.82264 (17)	0.0959 (2)	0.61692 (16)	0.0436 (6)	
N2	0.73604 (15)	0.3018 (2)	0.53140 (15)	0.0349 (5)	
N3	0.79833 (15)	−0.0104 (2)	0.45376 (16)	0.0407 (6)	
N4	0.65620 (15)	0.1222 (2)	0.40350 (15)	0.0362 (5)	
O1	0.50092 (17)	0.8086 (3)	0.19076 (18)	0.0680 (7)	
H1B	0.5193	0.8540	0.2424	0.082*	
O11	0.44953 (15)	0.9508 (2)	0.40876 (14)	0.0556 (6)	
O12	0.43016 (16)	0.9986 (3)	0.27602 (17)	0.0634 (7)	
O13	0.36408 (16)	0.8190 (2)	0.30031 (17)	0.0628 (7)	
O14	0.31790 (16)	1.0195 (3)	0.30381 (17)	0.0639 (7)	

### Atomic displacement parameters (Å^2^)

**Table:**

	*U*^11^	*U*^22^	*U*^33^	*U*^12^	*U*^13^	*U*^23^
C1	0.0486 (18)	0.0393 (18)	0.0505 (19)	0.0061 (15)	0.0116 (16)	0.0060 (15)
C2	0.0488 (18)	0.0376 (18)	0.0486 (19)	0.0091 (14)	0.0095 (15)	0.0165 (15)
C3	0.0494 (19)	0.061 (2)	0.0385 (17)	0.0044 (17)	0.0126 (15)	0.0105 (16)
C4	0.0482 (18)	0.0468 (19)	0.0387 (17)	0.0063 (15)	0.0134 (15)	0.0055 (14)
C5	0.0362 (15)	0.0360 (16)	0.0380 (15)	0.0020 (12)	0.0203 (13)	0.0036 (12)
C6	0.0468 (17)	0.0354 (17)	0.0341 (15)	0.0025 (13)	0.0163 (13)	0.0003 (12)
C7	0.0338 (14)	0.0349 (16)	0.0345 (15)	−0.0009 (12)	0.0114 (12)	0.0020 (12)
C8	0.0361 (14)	0.0320 (15)	0.0336 (15)	0.0005 (12)	0.0114 (12)	0.0059 (12)
C9	0.0452 (17)	0.0414 (17)	0.0375 (16)	−0.0043 (14)	0.0144 (14)	0.0005 (13)
C10	0.055 (2)	0.0382 (18)	0.0450 (18)	−0.0043 (15)	0.0140 (16)	−0.0050 (15)
C11	0.057 (2)	0.0370 (18)	0.0508 (19)	0.0074 (15)	0.0175 (17)	0.0027 (15)
C12	0.0476 (19)	0.0454 (19)	0.053 (2)	0.0141 (16)	0.0103 (16)	0.0078 (16)
C13	0.0472 (17)	0.0385 (17)	0.0379 (16)	−0.0009 (14)	0.0132 (14)	−0.0007 (13)
C14	0.0478 (18)	0.0432 (19)	0.054 (2)	0.0122 (15)	0.0150 (16)	−0.0007 (15)
C15	0.0447 (18)	0.056 (2)	0.055 (2)	0.0162 (16)	0.0160 (16)	0.0022 (17)
C16	0.054 (2)	0.0446 (19)	0.053 (2)	0.0180 (16)	0.0161 (17)	0.0082 (16)
C17	0.0456 (18)	0.0356 (17)	0.060 (2)	0.0038 (14)	0.0153 (16)	0.0067 (15)
C18	0.0410 (16)	0.0374 (17)	0.0469 (18)	0.0051 (13)	0.0154 (14)	0.0054 (14)
C19	0.0388 (16)	0.0300 (15)	0.0464 (18)	−0.0062 (12)	0.0115 (14)	−0.0010 (13)
C20	0.0447 (17)	0.0391 (17)	0.0400 (16)	−0.0047 (13)	0.0186 (14)	−0.0091 (13)
C21	0.0381 (15)	0.0504 (19)	0.0300 (15)	0.0026 (14)	0.0100 (13)	−0.0046 (13)
C22	0.0435 (18)	0.061 (2)	0.052 (2)	0.0088 (16)	0.0112 (16)	0.0169 (18)
C23	0.059 (2)	0.055 (2)	0.051 (2)	0.0086 (18)	0.0198 (18)	0.0135 (17)
C24	0.066 (2)	0.052 (2)	0.052 (2)	−0.0044 (18)	0.0199 (18)	0.0146 (17)
C25	0.052 (2)	0.053 (2)	0.053 (2)	−0.0111 (16)	0.0163 (17)	0.0013 (17)
C26	0.0442 (18)	0.056 (2)	0.0389 (17)	0.0007 (15)	0.0112 (14)	−0.0035 (15)
C27	0.0394 (16)	0.0392 (16)	0.0386 (16)	0.0036 (13)	0.0156 (13)	0.0075 (13)
C28	0.0287 (14)	0.0338 (16)	0.0509 (19)	−0.0001 (12)	0.0101 (13)	−0.0057 (13)
C29	0.073 (3)	0.061 (3)	0.063 (3)	−0.013 (2)	0.021 (2)	−0.010 (2)
Cl1	0.0397 (4)	0.0399 (4)	0.0418 (4)	0.0014 (3)	0.0154 (3)	0.0004 (3)
Cl2	0.0352 (4)	0.0541 (5)	0.0591 (5)	−0.0073 (3)	0.0146 (4)	0.0039 (4)
Cu1	0.03061 (19)	0.0358 (2)	0.0403 (2)	−0.00030 (15)	0.01249 (15)	−0.00317 (16)
N1	0.0446 (14)	0.0358 (14)	0.0408 (14)	0.0050 (11)	0.0126 (12)	0.0044 (11)
N2	0.0360 (12)	0.0325 (13)	0.0308 (12)	−0.0019 (10)	0.0114 (10)	−0.0002 (10)
N3	0.0369 (13)	0.0390 (14)	0.0406 (14)	0.0019 (11)	0.0139 (11)	−0.0011 (11)
N4	0.0318 (12)	0.0310 (12)	0.0402 (13)	−0.0034 (10)	0.0126 (11)	−0.0011 (10)
O1	0.0582 (15)	0.0594 (16)	0.0646 (16)	0.0164 (13)	0.0113 (13)	0.0211 (14)
O11	0.0487 (13)	0.0586 (15)	0.0453 (13)	−0.0095 (11)	0.0109 (11)	−0.0012 (11)
O12	0.0546 (14)	0.0589 (16)	0.0616 (16)	0.0028 (12)	0.0153 (13)	0.0166 (13)
O13	0.0516 (14)	0.0526 (15)	0.0672 (16)	−0.0052 (11)	0.0145 (13)	−0.0131 (13)
O14	0.0539 (14)	0.0623 (17)	0.0607 (16)	0.0141 (13)	0.0151 (13)	0.0054 (13)

### Geometric parameters (Å, º)

**Table:**

C1—N1	1.337 (4)	C19—N4	1.483 (4)
C1—C2	1.369 (5)	C19—H19A	0.9700
C1—H1	0.9300	C19—H19B	0.9700
C2—C3	1.370 (5)	C20—C21	1.502 (5)
C2—H2	0.9300	C20—N4	1.504 (4)
C3—C4	1.373 (5)	C20—H20A	0.9700
C3—H3	0.9300	C20—H20B	0.9700
C4—C5	1.378 (4)	C21—C26	1.377 (5)
C4—H4	0.9300	C21—C22	1.383 (5)
C5—N1	1.340 (4)	C22—C23	1.400 (5)
C5—C6	1.504 (4)	C22—H22	0.9300
C6—N2	1.476 (4)	C23—C24	1.347 (5)
C6—H6A	0.9700	C23—H23	0.9300
C6—H6B	0.9700	C24—C25	1.367 (5)
C7—N2	1.486 (4)	C24—H24	0.9300
C7—C8	1.518 (4)	C25—C26	1.368 (5)
C7—H7A	0.9700	C25—H25	0.9300
C7—H7B	0.9700	C26—H26	0.9300
C8—C9	1.379 (4)	C27—N2	1.496 (4)
C8—C13	1.394 (4)	C27—C28	1.505 (4)
C9—C10	1.393 (5)	C27—H27A	0.9700
C9—H9	0.9300	C27—H27B	0.9700
C10—C11	1.368 (5)	C28—N4	1.496 (4)
C10—H10	0.9300	C28—H28A	0.9700
C11—C12	1.367 (5)	C28—H28B	0.9700
C11—H11	0.9300	C29—O1	1.414 (5)
C12—C13	1.373 (5)	C29—H29A	0.9600
C12—H12	0.9300	C29—H29B	0.9600
C13—H13	0.9300	C29—H29C	0.9600
C14—N3	1.336 (4)	Cl1—O11	1.409 (2)
C14—C15	1.376 (5)	Cl1—O13	1.419 (3)
C14—H14	0.9300	Cl1—O14	1.423 (3)
C15—C16	1.359 (5)	Cl1—O12	1.425 (3)
C15—H15	0.9300	Cu1—N3	1.983 (3)
C16—C17	1.394 (5)	Cu1—N2	2.022 (2)
C16—H16	0.9300	Cu1—N4	2.087 (2)
C17—C18	1.359 (5)	Cu1—N1	2.152 (3)
C17—H17	0.9300	Cu1—Cl2	2.2830 (9)
C18—N3	1.359 (4)	O1—H1B	0.9600
C18—C19	1.500 (4)		
			
N1—C1—C2	123.2 (3)	C26—C21—C22	117.8 (3)
N1—C1—H1	118.4	C26—C21—C20	121.0 (3)
C2—C1—H1	118.4	C22—C21—C20	121.2 (3)
C1—C2—C3	118.5 (3)	C21—C22—C23	120.6 (3)
C1—C2—H2	120.7	C21—C22—H22	119.7
C3—C2—H2	120.7	C23—C22—H22	119.7
C2—C3—C4	119.4 (3)	C24—C23—C22	119.6 (3)
C2—C3—H3	120.3	C24—C23—H23	120.2
C4—C3—H3	120.3	C22—C23—H23	120.2
C3—C4—C5	118.9 (3)	C23—C24—C25	120.6 (4)
C3—C4—H4	120.5	C23—C24—H24	119.7
C5—C4—H4	120.5	C25—C24—H24	119.7
N1—C5—C4	122.2 (3)	C24—C25—C26	120.1 (3)
N1—C5—C6	115.8 (3)	C24—C25—H25	119.9
C4—C5—C6	122.0 (3)	C26—C25—H25	119.9
N2—C6—C5	112.0 (2)	C25—C26—C21	121.3 (3)
N2—C6—H6A	109.2	C25—C26—H26	119.3
C5—C6—H6A	109.2	C21—C26—H26	119.3
N2—C6—H6B	109.2	N2—C27—C28	110.1 (2)
C5—C6—H6B	109.2	N2—C27—H27A	109.6
H6A—C6—H6B	107.9	C28—C27—H27A	109.6
N2—C7—C8	116.8 (2)	N2—C27—H27B	109.6
N2—C7—H7A	108.1	C28—C27—H27B	109.6
C8—C7—H7A	108.1	H27A—C27—H27B	108.1
N2—C7—H7B	108.1	N4—C28—C27	111.2 (2)
C8—C7—H7B	108.1	N4—C28—H28A	109.4
H7A—C7—H7B	107.3	C27—C28—H28A	109.4
C9—C8—C13	118.2 (3)	N4—C28—H28B	109.4
C9—C8—C7	120.8 (3)	C27—C28—H28B	109.4
C13—C8—C7	120.8 (3)	H28A—C28—H28B	108.0
C8—C9—C10	120.2 (3)	O1—C29—H29A	109.5
C8—C9—H9	119.9	O1—C29—H29B	109.5
C10—C9—H9	119.9	H29A—C29—H29B	109.5
C11—C10—C9	120.6 (3)	O1—C29—H29C	109.5
C11—C10—H10	119.7	H29A—C29—H29C	109.5
C9—C10—H10	119.7	H29B—C29—H29C	109.5
C12—C11—C10	119.7 (3)	O11—Cl1—O13	108.66 (16)
C12—C11—H11	120.2	O11—Cl1—O14	110.26 (16)
C10—C11—H11	120.2	O13—Cl1—O14	107.23 (17)
C11—C12—C13	120.3 (3)	O11—Cl1—O12	109.60 (15)
C11—C12—H12	119.9	O13—Cl1—O12	113.89 (17)
C13—C12—H12	119.9	O14—Cl1—O12	107.14 (16)
C12—C13—C8	121.1 (3)	N3—Cu1—N2	161.01 (10)
C12—C13—H13	119.4	N3—Cu1—N4	81.13 (10)
C8—C13—H13	119.4	N2—Cu1—N4	86.06 (10)
N3—C14—C15	122.1 (3)	N3—Cu1—N1	92.73 (10)
N3—C14—H14	118.9	N2—Cu1—N1	80.29 (10)
C15—C14—H14	118.9	N4—Cu1—N1	115.89 (10)
C16—C15—C14	118.1 (3)	N3—Cu1—Cl2	99.64 (8)
C16—C15—H15	120.9	N2—Cu1—Cl2	99.03 (7)
C14—C15—H15	120.9	N4—Cu1—Cl2	142.95 (7)
C15—C16—C17	120.6 (3)	N1—Cu1—Cl2	101.12 (8)
C15—C16—H16	119.7	C1—N1—C5	117.8 (3)
C17—C16—H16	119.7	C1—N1—Cu1	130.9 (2)
C18—C17—C16	118.5 (3)	C5—N1—Cu1	111.08 (19)
C18—C17—H17	120.7	C6—N2—C7	110.6 (2)
C16—C17—H17	120.7	C6—N2—C27	109.8 (2)
N3—C18—C17	121.2 (3)	C7—N2—C27	113.4 (2)
N3—C18—C19	114.4 (3)	C6—N2—Cu1	107.97 (18)
C17—C18—C19	124.4 (3)	C7—N2—Cu1	112.35 (17)
N4—C19—C18	108.3 (3)	C27—N2—Cu1	102.29 (18)
N4—C19—H19A	110.0	C14—N3—C18	119.4 (3)
C18—C19—H19A	110.0	C14—N3—Cu1	128.9 (2)
N4—C19—H19B	110.0	C18—N3—Cu1	111.4 (2)
C18—C19—H19B	110.0	C19—N4—C28	111.5 (2)
H19A—C19—H19B	108.4	C19—N4—C20	108.3 (2)
C21—C20—N4	114.1 (2)	C28—N4—C20	110.0 (2)
C21—C20—H20A	108.7	C19—N4—Cu1	99.18 (17)
N4—C20—H20A	108.7	C28—N4—Cu1	106.68 (17)
C21—C20—H20B	108.7	C20—N4—Cu1	120.60 (18)
N4—C20—H20B	108.7	C29—O1—H1B	109.3
H20A—C20—H20B	107.6		

### Hydrogen-bond geometry (Å, º)

**Table:**

*D*—H···*A*	*D*—H	H···*A*	*D*···*A*	*D*—H···*A*
O1—H1*B*···O12	0.96	2.48	3.140 (4)	126
